# Microleakage of an Enhanced Resin-Modified Glass Ionomer Restorative Material in Primary Molars

**Published:** 2018-07

**Authors:** Baharan Ranjbar Omidi, Fatemeh Ferdowsizadeh Naeini, Hajar Dehghan, Parvin Tamiz, Maryam Mohammadi Savadroodbari, Razieh Jabbarian

**Affiliations:** 1 Assistant Professor, Department of Operative Dentistry, Dental Caries Prevention Research Center, Qazvin University of Medical Sciences, Qazvin, Iran; 2 Assistant Professor, Department of Pediatric Dentistry, Faculty of Dentistry, Kashan University of Medical Sciences, Kashan, Iran; 3 Assistant Professor, Department of Pediatric Dentistry, Faculty of Dentistry, Qazvin University of Medical Sciences, Qazvin, Iran; 4 Postgraduate Student, Department of Orthodontics, Faculty of Dentistry, Qazvin University of Medical Sciences, Qazvin, Iran; 5 Assistant Professor, Department of Operative Dentistry, Faculty of Dentistry, Alborz University of Medical Sciences, Karaj, Iran; 6 Postgraduate Student, Department of Pediatric Dentistry, Faculty of Dentistry, Qazvin University of Medical Sciences, Qazvin, Iran

**Keywords:** Resin-Modified Glass Ionomer, Molar, Primary Dentition, Dental Leakage

## Abstract

**Objectives:**

Resin composites, glass ionomers (GIs), or a combination of these materials have gradually replaced silver amalgam in pediatric dentistry. The purpose of this study was to compare the microleakage of Class II (box only) cavity restorations with ACTIVA Bioactive Restorative Glass, resin-modified GI (RMGI), and composite in primary molars.

**Materials and Methods:**

A total of 65 primary molars with at least one intact proximal surface were selected in this in-vitro study. After debridement of each tooth, Class II (box only) cavities were prepared. Based on the type of the restorative material and the application of etching and bonding adhesives, the samples were categorized into five groups: (1) composite; (2) RMGI (Fuji II LC)+conditioner; (3) RMGI (Fuji II LC); (4) enhanced RMGI (ACTIVA Bioactive Restorative Glass)+etching/bonding; and (5) ACTIVA Bioactive Restorative Glass. The restored teeth were thermocycled for 2000 cycles. After embedding in an acrylic resin, the degree of dye penetration at axial and gingival walls was assessed using a stereomicroscope. The data were statistically analyzed by analysis of variance (ANOVA) and Tukey’s test.

**Results:**

Resin-based composite (RBC) Z250 showed the least microleakage, while RMGI showed maximum microleakage at axial walls. The mean degree of microleakage at gingival margins was the lowest in RBC Z250 and ACTIVA+etching/bonding groups and the highest in RMGI+conditioner and RMGI groups.

**Conclusions:**

The microleakage of ACTIVA Bioactive Restorative material in the absence or presence of etching and bonding could be comparable to the microleakage of composites.

## INTRODUCTION

In pediatric dentistry, the most common materials in the restoration of primary molars are composites and other resin-based (RB) materials, glass ionomers (GIs), silver amalgam alloys, and stainless steel crowns. Resin composites, GIs, or a combination of both have gradually replaced silver amalgam in pediatric restorative dentistry [1]. Considering the manufacturers’ suggestions for the use of posterior composites with less microleakage and a wear resistance comparable to that of amalgams, these materials have been recently applied for the restoration of primary molars in small Class I and II cavities in an attempt to reduce the possible damage resulting from the presence of mercury in amalgams [2].

Considering the high technical sensitivity and time-consuming application of composite restorations, GI cements are proper options for the restoration of primary molars. In spite of their chemical bonds to tooth structure and fluoride release, GI cements exhibit poorer mechanical features in comparison with composites, which limits their application in stress-bearing areas [3]. To enhance the mechanical properties of GIs, their constituents have been modified. Comparatively, resin-modified GIs (RMGIs) have a longer working time, faster setting, higher early strength, and improved appearance and translucency [4]. However, the mechanical properties of RMGIs are not similar to those of composites [5]. Continuous development of material sciences has resulted in the introduction of bioactive restorative materials. These materials can activate a mechanism for tissue repair or synthesis and elicit a response from teeth [6]. Recently, a novel bioactive restorative material has been developed known as ACTIVA Bioactive Restorative Glass (Pulpdent Corp., Watertown, MA, USA). According to the manufacturer, these bioactive products contain a bioactive ionic resin matrix, shock absorbing resin components, and reactive GI fillers that imitate the physical and chemical properties of natural teeth [7].

The ACTIVA products comprise an enhanced RMGI with a blend of diurethane monomers modified by the insertion of a hydrogenated polybutadiene (a synthetic rubber) and methacrylate-based monomers. The added resin monomers are claimed to improve wear resistance, fracture, and marginal chipping [8].

These products include bioactive fillers, which mimic the physical and chemical properties of natural teeth. They actively participate in a dynamic system of ion exchange with the saliva and tooth structure [7]. In addition, they can release and recharge with calcium, phosphate and more fluoride than GIs and continuously react to pH changes in the mouth. They can also form a chemical bond to teeth and seal the cavities against bacterial microleakage [9–11].

According to the manufacturers, ACTIVA bioactive products are strong, esthetic, and long-lasting restorative materials that can replace composites which have the same properties but lack potential bioactive components. They can also replace GIs, which are bioactive but have poor esthetics and poor physical properties [7]. Previous studies have shown that ACTIVA products have physical characteristics which closely resemble the strength and wear resistance of RB composites, although they do not contain bisphenol A or its derivatives [10].

Microleakage is the most common cause of failure in almost all restorative materials since it results in secondary caries and pulpal irritation [12]. Therefore, the purpose of this in-vitro study was to evaluate the microleakage of a novel GI, known as ACTIVA Bioactive Restorative Glass, and to compare it with RMGI and resin composites in Class II (box only) restorations of primary molars.

## MATERIALS AND METHODS

In this in-vitro experimental study, a total of 65 primary molars with at least one intact proximal surface were selected. This study has been approved by the Ethics Committee of School of Dentistry, Qazvin University of Medical Sciences (IR.QUMS.REC.1395.19). Hypoplastic or hypocalcified teeth, as well as the teeth with caries involving more than one-fourth of the occlusal surface, were excluded from the study. The teeth were observed under a stereomicroscope (MBC-2, St Petersburg, Russia; 10× magnification) to ensure the absence of any cracks or fracture lines.

Following dental debridement, the teeth were placed in distilled water at room temperature (25°C). A total of 65 Class II (box only) cavities were prepared in intact dental surfaces. The cavity dimensions were 3.0 mm buccolingually, 1.5 mm mesiodistally, and 3.0 mm occlusogingivally, and they were prepared by the use of high-speed fissure diamond burs (#008 Diamir, Italy) under constant water cooling. The cavity dimensions were verified using a digital caliper (Mitutoyo Corp., Tokyo, Japan; accuracy of ±0.25 mm). The bur was replaced after every five preparations.

Next, the teeth were randomly divided into five groups according to the type of restorative materials and use of conditioning agents, as listed below. The commercial names, compositions, and manufacturers of the materials used in this study are listed in Table 1.

**Table 1:** Commercial names, compositions, and manufacturers of the materials

**Dental material**	**Composition**
Bonding Agent: Adper™ Scotchbond™ 1 XT Adhesive (3M ESPE, St. Paul, MN, USA)	Bis-GMA, HEMA, Bisphenol A glycerolate dimethacrylate, copolymer of polyacrylic and polyitaconic acids, water, ethanol
Composite: Filtek Z250 (3M ESPE, St. Paul, MN, USA)	Bis-GMA, UDMA, Bis-EMA, TEGDMA, Zirconia, silica (0.01–3.5 μm, 75 wt%)
RMGI: Fuji II LC (GC Corp., Tokyo, Japan)	Liquid: Distilled water: 20–30% Polyacrylic acid: 20–30% HEMA: 30–35% UDMA<10 Camphorquinone<1 Powder: fluoroaluminosilicate glass
Reinforced RMGI: ACTIVA Bioactive Restorative Glass (Pulpdent Corp., Watertown, MA, USA)	Blend of diurethane and other methacrylates with modified polyacrylic acid (44.6%), amorphous silica (6.7%), and sodium fluoride (0.75%)

RMGI=Resin-Modified Glass Ionomer, HEMA=2-hydroxyethyl methacrylate, UDMA=Urethane dimethacrylate, Bis-GMA=Bisphenol A glycol dimethacrylate, TEGDMA=Triethylene glycol dimethacrylate, Bis-EMA=Ethoxylated bisphenol A glycol dimethacrylate

**Group 1**: 38% phosphoric acid (Pulpdent Corp., Watertown, MA, USA) was first applied to enamel margins for five seconds and then to the dentin for 15 seconds [13]. Following that, the samples were rinsed with water for 15 seconds and were gently dried using an air spray. Two bonding layers of Adper Single Bond Adhesive (3M ESPE, St. Paul, MN, USA) were used in the cavities.

The layers were gently air-blown and light-cured (Monitex, BlueLEX TM GT-1200, New Taipei, Taiwan; 800 mW/cm^2^) for 20 seconds. The light intensity was measured with a light-emitting diode (LED) curing radiometer (Wireless LED Dental Curing Light Lamp 1800MW, Kerr, USA). Afterwards, the composite (Filtek Z250, 3M ESPE, St. Paul, MN, USA) was placed inside the cavities in 2-mm incremental layers, and each layer was polymerized for 40 seconds. The specimens were stored in distilled water for 24 hours at 37°C before being polished by polishing disks (Shofu, Kyoto, Japan) under continuous water spray.

**Group 2**: The acrylic acid conditioner (20%; GC Corp., Tokyo, Japan) was applied to the cavities for 10 seconds. The cavities were subsequently rinsed, and moisture was removed by a cotton roll. RMGI (Fuji II LC, GC Corp., Tokyo, Japan) was mixed on a glass slab according to the manufacturer’s instructions. The material was placed in tooth cavities and was light-cured for 20 seconds. The specimens were kept in distilled water for 24 hours at 37°C before being polished in the same manner as group 1.

**Group 3**: The RMGI was mixed based on the manufacturer’ instruction and was placed in clean cavities without conditioning the teeth. After storage in distilled water at 37°C for 24 hours, the specimens were polished.

**Group 4**: Dental cavities were etched using 38% phosphoric acid for 10 seconds. Then, the cavities were rinsed using a water spray for 20 seconds, and excess moisture was removed using a low-pressure air spray. Subsequently, two layers of bonding agent (Adper Single Bond Adhesive) were placed in each cavity and were light-cured for 20 seconds. Later, ACTIVA was injected into each cavity using a syringe, according to the manufacturer’s instruction. The samples were left for 20 seconds to allow primary acid-base reactions; afterwards, they were light-cured for 20 seconds. The specimens were stored in distilled water at 37°C for 24 hours before being polished.

**Group 5**: The specimens in this group were restored with ACTIVA in the same manner as group 4; however, the teeth were not conditioned before the application of the restorative material. Finally, the samples were thermocycled (Dorsa, Malek Teb, Tehran, Iran) for 2000 cycles (5±2°C and 55±2°C) with immersion for 30 seconds [11,14]. An interval of 30 seconds was set between the two immersion periods.

### Preparation of teeth for Dye Penetration Test:

The apex and bifurcation of each tooth as well as the root which had already initiated the process of physiological absorption were sealed with a flowable composite (Diadent Inc., Chongchong Buk Do, Korea). Additionally, all dental surfaces were sealed with two layers of nail polish, except for 1–1.5 mm margins around the cavities. The samples were immersed in a 1M silver nitrate solution (17g in 100cc of distilled water; Ranbaxy Laboratories Ltd., Delhi, India) at room temperature for 24 hours. They were later rinsed with water for five minutes. Afterwards, the samples were placed in a photochemical developer for exposure to fluorescent light for 12 hours; they were subsequently rinsed for five minutes.

The specimens were cut longitudinally in a mesiodistal direction through the restoration center using a high-speed diamond saw (Nemopars, Iran) with water cooling; consequently, two samples were obtained from each cavity.

The degree of microleakage was determined by an operator blinded to the samples using a stereomicroscope (EZ4D; Leica Microsystems GmbH, Wetzlar, Germany) at 40× magnification in order to determine the extent of dye penetration at axial and gingival margins.

The images acquired by the stereomicroscope were analyzed by the Intuitive LAS EZ 1.6.0 software (Leica Microsystems GmbH, Wetzlar, Germany). The degree of dye penetration into each wall was recorded in micrometers (μm) and was divided by the width of each wall to determine the percentage of penetration [4, 15]. The data were analyzed using SPSS version 21 (SPSS Inc., Chicago, IL, USA). Analysis of variance (ANOVA), paired t-test, and Tukey’s test were used for data analysis, and the level of significance was set at P=0.05.

## RESULTS

Tables 2 and 3 demonstrate the percentage of leakage at axial walls and gingival margins in the groups.

**Table 2:** Mean percentage (%) of dye penetration at axial walls in the groups

**Group**	**Mean**	**Standard deviation**	**Min**	**Max**
Composite (Filtek Z250)	5.2	1.2	0	32
RMGI Fuji II LC+conditioner	25.6	3.2	0	47
RMGI Fuji II LC	30.5	3.9	0	76
ACTIVA+etch/bond	12.9	3.7	0	62
ACTIVA	9.8	3.3	0	59

RMGI=Resin-Modified Glass Ionomer

**Table 3:** Mean percentage (%) of dye penetration at gingival margins in the groups

**Group**	**Mean**	**Standard deviation**	**Min**	**Max**
Composite (Filtek Z250)	31.8	6.7	0	88
RMGI Fuji II LC+conditioner	62.4	5.2	0	98
RMGI Fuji II LC	59.9	4.2	5	90
ACTIVA+etch/bond	30.3	5.1	0	77
ACTIVA	46.9	6.04	0	95

RMGI=Resin-Modified Glass Ionomer

The results of one-way ANOVA revealed significant differences between the groups at axial walls (P<0.001). Minimum penetration was reported in RBC Z250 group, while maximum penetration was observed in the RMGI group without conditioning (Table 2).

Pairwise comparisons of the groups revealed that the difference between groups 2 (RMGI+conditioner) and 3 (RMGI) was not significant. Moreover, there was no significant difference between RBC Z250 and ACTIVA groups or between RBC Z250 and ACTIVA+etching/bonding groups. In addition, the analysis showed no significant difference between the ACTIVA and ACTIVA+etching/bonding groups (P>0.05; Table 4).

**Table 4:** Pairwise comparisons of the mean percentage (%) of dye penetration at axial walls and gingival margins between the groups (post-hoc Tukey’s test)

**Groups**	**P-value (axial walls)**	**P-value (gingival walls)**
RBC Z250 and RMGI+conditioner	0.001	0.002
RBC Z250 and RMGI	<0.001	0.006
RBC Z250 and ACTIVA+etch/bond	0.50	0.99
RBC Z250 and ACTIVA	0.86	0.03
RMGI+conditioner and RMGI	0.84	0.98
RMGI+conditioner and ACTIVA+etch/bond	0.047	0.001
RMGI+conditioner and ACTIVA	0.008	0.04
RMGI and ACTIVA+etch/bond	0.003	0.002
RMGI and ACTIVA	<0.001	0.043
ACTIVA+etch/bond and ACTIVA	0.96	0.02

RBC=Resin-Based Composite, RMGI=Resin-Modified Glass Ionomer

The analysis of the average dye penetration into the gingival margins revealed a significant difference between the groups (P<0.001) with the maximum microleakage in RMGI+conditioner and RMGI groups, and the minimum microleakage in RBC Z250 and ACTIVA+etching/bonding groups (Table 3).

A closer inspection showed that microleakage was significantly higher in RMGI+conditioner and RMGI groups compared to the ACTIVA group. Also, the microleakage in the ACTIVA group was significantly greater than that of RBC Z250 and ACTIVA+etching/bonding groups (P<0.05; Table 4). The result of paired t-test showed that the average dye penetration in each group at gingival margins was significantly greater than the microleakage at axial walls (P<0.05).

## DISCUSSION

Restoration of decayed primary teeth requires materials that are fast-setting and less technique-sensitive. Therefore, restorative materials which require fewer application steps and thus reduce the risk of contamination and treatment time are the focus of research [16]. A new bioactive material, known as ACTIVA Bioactive Restorative Glass, has been recently introduced. The manufacturer believes that this product has the advantages of both composites and RMGIs [10], and therefore, it can be an ideal material in pediatric dentistry. As there are only a few studies on this novel material, in the current study, we aimed to compare its microleakage with that of RMGI and composite (Filtek Z250) in primary molars. In order to simulate the invivo aging of materials, thermocycling is used as the standard protocol in the restorative literature when evaluating bonded materials [3]. Nelsen et al [17] showed that oral temperature rises to 60°C within a few seconds of having a hot drink and reaches to as low as 4°C after having a cold drink [17]. According to previous studies, we used 2000 thermal cycles at 5°C and 55°C for the process of thermocycling [11,18].

To evaluate the microleakage of restorations, different methods have been used including dye penetration, air pressure, radioactive isotopes, and scanning electron microscopy (SEM). Dye penetration is considered a simple method as the dye penetrates successfully into the flaws and crevices of the test object [19]. One of the most commonly used dyes in microleakage tests is silver nitrate which has a higher penetration into the microgaps between the restorative material and tooth structure compared to fuchsine and methylene blue [13].

The present study performed the quantitative microleakage evaluation method instead of the conventional subjective scoring. The advantage of this approach, when compared to the qualitative scoring method, is that it discards the need for scoring by separate evaluators and for consensus scoring in borderline cases. It also reduces the need for statistical procedures regarding the inter-examiner reliability [20, 21].

The present study showed that microleakage in all groups was higher at gingival margins compared to axial walls (P<0.05). Since the margins of the occlusal enamel were not removed during the preparation of Class II cavities, and since the occlusal enamel had a greater thickness than gingival margins, more microleakage was observed at gingival margins in all the studied groups. This finding is consistent with the results reported by Siddique and Karkare [22], Gerdolle et al [23], Hussein et al [24], Eronat et al [25], Abd EL Halim and Zaki [3], Pontes et al [26], and Shih [18]. These studies showed that microleakage at gingival margins was greater than that at axial walls, irrespective of the restorative material. Eronat et al [25] attributed this difference to the strength of bonding between the restorative material, enamel, and dentin, arguing that the higher mineral content of the enamel leads to a stronger bond.

In addition, Brown et al [27] in their study of gingival margins, reported some cracks in the dentin as well as a thinner enamel, which could affect microleakage. With regard to the axial margin, the current study showed a significant difference between the groups. Based on the results, the maximum and minimum microleakage at axial walls were observed in RMGI and RBC Z250 groups, respectively. In other groups, the degree of microleakage was in the following descending order: RMGI+conditioner, ACTIVA+etching/bonding, and ACTIVA. These findings show that microleakage of the reinforced RMGI (ACTIVA) is comparable to that of composites (P>0.05), as claimed by the manufacturer [28].

As for the microleakage at gingival margins, the findings showed that the microleakage in RMGI+conditioner and RMGI groups was significantly higher than that of the ACTIVA group. Also, the microleakage in the ACTIVA group was significantly greater than that of RBC Z250 and ACTIVA+etching/bonding groups (P<0.05).

Overall, the microleakage of RMGI Fuji II LC with and without the conditioner was significantly greater than that of resin composite Z250 (P<0.05).

The greater microleakage of RMGI Fuji II LC can be attributed to the mechanism through which this material bonds to dental structures. The setting of RMGI is essentially achieved by an acid-base reaction. A polymerization reaction also occurs with 2-hydroxyethyl methacrylate (HEMA) and urethane-dimethacrylate (UDMA) monomers of the resin matrix, producing additional shrinkage. The weaker bond strength of RMGI to both enamel and dentin could explain the high level of leakage [23]. Additionally, Mitra et al [29] reported that since the bonding strength between RMGI and tooth structure is weak, the stress resulting from polymerization shrinkage and dimensional changes can compromise this bonding and increase microleakage.

Another possible explanation is that the mixing procedure during preparation can result in the formation of bubbles, which in turn contributes to leakage [30]. In addition, the lower filler content of RMGI indicates a higher resin content, which increases the polymerization shrinkage and consequently the microleakage [25].

These findings are in agreement with a study by Nematollahi et al [31]; they attributed the lower degree of microleakage in the composite (Filtek Z250) to the lower degree of polymerization shrinkage in comparison with RMGI [31]. Khoroushi et al [32] also compared the microleakage of Fuji II LC with another type of composite and reported similar results.

In another study, Gerdolle et al [23] found the highest degree of shrinkage in Fuji II LC restorative versus the compomer and composite. They also stated that the higher polymerization shrinkage in RMGI could be one of the factors causing the higher degree of microleakage in RMGI [23].

In contrast, in studies by Diwanji et al [19], Singla et al [30], and Rekha et al [33], a low degree of microleakage was reported for RMGI (Fuji II LC). Although these studies compared RMGI with other types of GIs and compomers, the sample storage environment and the timing of storage were different from those of the present study. It has been stated that the good sealing ability of light-cured resin-reinforced restorative cements can be explained by water sorption, which results in the subsequent hygroscopic expansion of the material and decreases the marginal gap between the restoration and tooth [23, 30]. Different storing conditions in our study could lead to different levels of water absorption and consequently different amounts of microleakage. Our findings also showed a higher degree of microleakage in RMGI Fuji II LC with and without the conditioner in comparison with ACTIVA (P<0.05). The ACTIVA product has an ionic resin network and bioactive fillers which can further reduce the polymerization shrinkage [25]. The enhanced RMGIs already contain reactive, ion-releasing glasses, which in the broadest sense, render this class of biomaterials as “bioactive”. The active release of calcium, phosphate, and fluoride ions from ACTIVA Bioactive materials and their interactions with the dentin and enamel can benefit the longevity of the restoration [9]. However, as ACTIVA is a new material, we could not find any previous studies comparing its microleakage with that of RMGI. The present study also found that microleakage at axial walls was greater in ACTIVA+etching/bonding group compared to the ACTIVA group; however, the difference was not significant. Overall, since ACTIVA is an enhanced RMGI, it has three setting mechanisms similar to other types of RMGI: light-activated polymerization, chemically activated polymerization of the resin, and the acid-base reaction of the GI. The basic bonding mechanism involves ionic attraction of two carboxyl (COO^−^) groups in the cement to calcium (Ca^++^) in the enamel and dentin [3]. Therefore, ACTIVA products form micromechanical and chemical bonds with the tooth. On the other hand, ionic resins with an acidic nature can change the smear layer to some extent and form a strong bond. If etching and bonding are applied, only a micromechanical bond will be formed, while by removing calcium from dentin via etching, no chemical bond can be formed between GI and dentin [3]. The manufacturers recommend etching in non-retentive cavities [7], but it seems that the procedure of etching increases the microleakage. The present study found that microleakage of ACTIVA, with or without etching and bonding, is comparable to that of composites. This finding confirms the manufacturer’s claim. Unlike our study, Alkhudhairy and Ahmad [34] reported a moderate level of microleakage in ACTIVA Bioactive Restorative Glass in Class II (box only) cavities of maxillary premolars, which was higher than that of SureFil SDR^®^ composite (Dentsply, USA). The disagreement between the findings could be attributed to the differences in the type of teeth. It has been suggested that leakage in primary and permanent teeth may vary with each type of material; one may show a greater leakage in primary teeth, while another shows more leakage in permanent teeth [12]. Variations in the type of composites can also produce different results. It should be noted that the current study evaluated cavities above the cementoenamel junction (CEJ), and therefore, both gingival and axial walls contained enamel. However, in the study by Alkhudhairy and Ahmad [34], the cervical margin of the cavities was below the CEJ. Furthermore, our specimens were subjected to more thermocycles compared to those evaluated by Alkhudhairy and Ahmad [34].

## CONCLUSION

In the present study, none of the restorative materials were without microleakage. The highest degree of microleakage was observed in RMGI (Fuji II LC) group, either with or without conditioning. In almost all restorative materials, the microleakage at gingival margins was significantly greater than the microleakage at axial walls. In addition, the microleakage of the enhanced RMGI (ACTIVA Bioactive Restorative Glass) was comparable to that of Z250 resin composite.
